# Assessing the Knowledge, Attitude, and Practice of Healthcare Workers on the Herpes Zoster Vaccine in Saudi Arabia: A Cross-Sectional Study

**DOI:** 10.7759/cureus.77302

**Published:** 2025-01-11

**Authors:** Nouran M Moustafa, Norah F Alsaif, Esra Alsaeed, Alreem Alanezi, Amani Algarni, Lian Alkathery, Rania Mohamed

**Affiliations:** 1 Basic Medical Sciences, College of Medicine, Dar Al Uloom University, Riyadh, SAU; 2 Medical Microbiology and Immunology Department, Faculty of Medicine, Ain Shams University, Cairo, EGY; 3 College of Medicine, Dar Al Uloom University, Riyadh, SAU

**Keywords:** attitude, healthcare workers, knowledge, practice, shingles vaccine, zoster disease

## Abstract

Background and objectives: Herpes zoster (HZ), known as shingles disease, has several complications that greatly affect elderly and immunocompromised people. Despite evidence for HZ vaccine efficiency and cost-effectiveness, the uptake of the HZ vaccine is lower than that of other common adult vaccines. Strong recommendations by healthcare workers (HCWs) for the HZ vaccine could increase vaccine uptake.

Aim: This study aimed to investigate HCWs' knowledge, attitude, and practice of the recombinant zoster vaccine (RZV) in Saudi Arabia and identify gaps and barriers in vaccine recommendation.

Materials and methods: A cross-sectional, questionnaire-based study was conducted in different hospitals in Riyadh, Saudi Arabia, between December 2023 and March 2024. An electronic self-administered validated questionnaire that assesses the knowledge, attitude, and practice of HCWs regarding the HZ vaccine was sent to all hospital staff. The collected data were organized, entered on an Excel sheet, and statistically analyzed using SPSS software. Pearson's chi-square test and Monte Carlo exact tests were used for data analysis.

Results: A total of 309 HCWs responded to the survey. Family physicians, infectious diseases doctors, and dermatologists are essential to recommend vaccines in Saudi Arabia. In particular, 264 (86.4%) HCWs correctly identified the doses of RZV. However, 148 (47.9%) incorrectly believed that side effects from the first dose were a valid reason not to receive the second dose. Most HCWs (n = 235, 76.1) agreed that they need more direction on which immunocompromised patients are eligible for the RZV vaccine. Most physicians recommend the vaccine for patient groups mentioned in the Advisory Committee on Immunization Practices (ACIP) recommendations. However, 85 (27.5%) recommended the vaccine for healthy adults aged 18-49 years. Ninety-nine (32%) patients declined the vaccine as they did not think they were at risk for developing shingles and 73 (23.6%) declined due to fears of immediate side effects. Among the strategies recommended to increase vaccine uptake are distributing zoster vaccine pamphlets to patients and having a hotline or a website available to discuss zoster vaccination, which were recommended by 255 (82.5%) and 235 (76.1%) HCWs, respectively.

Conclusions: Physicians in Saudi Arabia are generally knowledgeable about the HZ vaccine but need more guidance about ACIP recommendations. The patient reactions to the first dose, the limited vaccine availability, and the perception that RZV has not been adequately studied in immunocompromised patients are among the barriers to vaccine recommendation. Pamphlets regarding the HZ vaccine and a hotline or a website available for discussion about the vaccine are recommended strategies to increase vaccine uptake.

## Introduction

Herpes zoster (HZ) also known as shingles is a reactivated form of varicella-zoster virus (VZV) that causes chickenpox. It is characterized by contagious painful vesicular eruptions affecting specific dermatomes, commonly lumbar and thoracic, which last for seven to 10 days [1]. HZ occurs exclusively in elderly and immunocompromised people with a previous history of chickenpox [2]. Some chronic diseases are also related to increased incidence of HZ (e.g., chronic obstructive pulmonary disease, diabetes) [3]. HZ has several complications that greatly affect patients. One of its common complications is post-herpetic neuralgia (PHN), which is characterized by severe neuropathic pain lasting for a minimum of three months after the rash onset. HZ ophthalmicus and HZ encephalitis are other complications that could also happen, ruin social activity, and interfere with the quality of patient life [4,5]. The latest research included HZ as one of the top 10 most costly skin diseases that increase the healthcare burden [6].

HZ therapeutic options mainly target pain relief and reducing complications [7]. Nevertheless, antiviral treatments such as acyclovir and anti-inflammatory drugs are only effective in decreasing the severity and duration of acute disease and do not reliably affect PHN [8]. The effective treatment of these conditions is usually vague and difficult, which makes prevention more convincing [9]. Preventive strategies are developed and considered the most efficient way to protect the health of patients, especially the elderly [10]. To prevent HZ and its complications, the US approved two vaccine products, namely, zoster vaccine live (ZVL) and recombinant zoster vaccine (RZV) [11].

Zoster vaccine live, known as Zostavax, is a single-dose live attenuated vaccine that was available for the elderly in the US from 2006 to 2017, yet Zostavax was not recommended for immunocompromised cases [12]. RZV, known as the Shingrix vaccine, is a two-dose vaccine that was approved in 2017 and exhibited greater and longer-lasting efficacy (>90% vs. 53%) [13]. It was recommended for use in immunocompetent and immunocompromised people ≥50 years to prevent VZ reactivation and decrease the severity of relapse [14]. It was recognized as effective and safe in immunocompromised cases having HIV, hematologic malignancies, and solid tumors, after renal and hematopoietic cell transplant [15]. Recently, in July 2021, the Food and Drug Administration (FDA) expanded the use of RZV in immunocompromised adults aged 18 years and older [16]. In October 2021, the Advisory Committee on Immunization Practices (ACIP) recommended RZV for immunocompromised adults aged 19 years and older [17]. The Centers for Disease Control and Prevention (CDC) recommended that the RZV vaccine be administered in two doses two to six months apart, even with a previous infection of HZ or with the last intake of Zostavax. Moreover, it is not compulsory to screen either through the laboratory or verbally for a previous infection with varicella. Immunocompromised patients or those who will be in an immunocompromised state should receive the two doses at ≥19 years. Although the second dose is typically administered after two to six months of the first dose, it can be received one or two months after the first dose in those people [18].

Despite the advances in the management of HZ infection, it remains a global serious public health concern. An estimated one million cases occur annually in the US among elderly individuals with an approximate ratio of four cases per 1,000 [19]. HZ burden and risk could be reduced by increasing HZ vaccine uptake [20]. Despite evidence for HZ vaccine efficiency and cost-effectiveness, the uptake of the HZ vaccine is lower than that of other common adult vaccines [21]. Global immunization rates demonstrate a lag for the HZ vaccine compared to annual influenza and pneumococcal vaccines [22]. Strong recommendations by healthcare workers (HCWs) for the HZ vaccine could reduce the lag [23]. Several studies have discussed HCW barriers to enhancing vaccine uptake [24]. Incomplete awareness of current HZ recommendations could also be considered. To our knowledge, there is a gap in the studies that evaluated the HCWs' knowledge of RZV. Thus, our goal is to investigate HCWs' knowledge, attitude, and practice (KAP) on the HZ vaccine, focusing on those caring for elderly and immunocompromised people. We aim also to identify gaps and barriers in vaccine recommendation and find opportunities to increase HZ vaccine knowledge and uptake in Saudi Arabia.

## Materials and methods

Study design

A quantitative, cross-sectional, questionnaire-based study was conducted among HCWs from different hospitals in Riyadh, Saudi Arabia, during the period between December 2023 and March 2024.

Study population and sample size

A self-administered electronic questionnaire was sent to the hospital's staff and the response was requested from those treating patients susceptible to HZ. Participants were notified that all responses would be anonymous to ensure the transparency of all data collected. 

OpenEpi (version 3.0) is used for sample size calculation considering a 95% confidence interval (CI), an anticipated frequency of 79%, and design effects of one [25].

Data collection tool

The questionnaire was adopted from other validated questionnaires used in different published studies [15,26,27,28]. it was reviewed, modified to fit with HCWs in Saudi Arabia, and then validated through a pilot study. Ethical approval (IRP) was provided by Dar Al Uloom University (IRB No.: HP-01-R-134-DAU-COM-24-11).

The questionnaire consisted of six sections (see Appendix). The first section asked about HCWs' demographics and practice characteristics. The second section involved questions about HCWs' knowledge regarding the shingles vaccine. The third section included HCWs' attitudes regarding zoster vaccine delivery. The fourth section covered the practice of HCWs and their recommendation of zoster vaccine to certain groups of patients including immunocompromised cases based on ACIP recommendations. The fifth section asked about experience with patients refusing the zoster vaccine. The sixth section covered barriers to recommending vaccination and strategies to overcome these barriers.

Piloting

The questionnaire was piloted by 20 HCWs (their results were also included in the final analysis), to assess the questions’ clarity, readability, and validity. According to their feedback, the questionnaire was amended as some items were changed to be clearer and applicable to HCWs. The overall questionnaire stability was measured by calculating Cronbach's alpha, which was 0.763.

Statistical analysis

The collected data were organized and entered on an Excel sheet (Microsoft Corpo., USA) and statistically analyzed using IBM SPSS Statistics for Windows, Version 25.0 (released 2017, IBM Corp., Armonk, NY). Qualitative data were presented by number and percent. 

The results were tabulated, grouped, and statistically analyzed using the Pearson chi-square test (χ²) to detect whether there is a significant association between different categorical variables, and when it was inappropriate, it was replaced by the Monte Carlo exact test. P-value was also used to indicate the level of significance (P ≤ 0.05: significant, and P < 0.001: highly significant).

## Results

A total of 309 HCWs responded to the survey. They included n = 157 (50.8%) males and n = 152 (40.5%) females whose ages ranged from 25 to 65 years old. Most of the participants’ age (n = 233, 75.5%) ranges from 25 to 44 years. They belonged to different specialties, including family physicians (n = 71, 23.0%), infectious diseases (n = 63, 20.4%), dermatologists (n = 48, 15.5%), general internists (n = 33, 10.7%), neurologists (n = 31, 10.0%), geriatricians (n = 22, 7.1%), oncologists (n = 17, 5.5%), and nurses (n = 24, 7.8%). The respondents' HCWs were consultants (n = 166, 53.7%), specialists (n = 51, 16.5%), general practitioners (n = 46, 14.9%), residents (n = 31, 10.0%), and fellows (n = 15, 4.9%). Regarding their experiences, n = 158 (51.1%) of them have a maximum of 10 years’ experience, n = 93 (30.1%) have 11-20 years’ experience, n = 40 (12.9%) have 21-30 years’ experience, and n = 18 (5.8%) have more than 30 years of experience. Most of the respondents' healthcare were from governmental hospitals (n = 177, 57.3%), while n = 58 (18.8%) from private hospitals, n = 48 (15.5%) from 1ry healthcare facilities, and n = 26 (8.4%) from clinics. The vaccine was available in n = 227 (73.5%) of settings (Table 1).

**Table 1:** Demographic data of the participants (n = 309)

	No.	%
Sex		
Male	157	50.8
Female	152	49.2
Age		
25-34 y	125	40.5
35-44 y	108	35.0
45-54 y	57	18.4
55-65 y	19	6.1
Specialty		
Family physician	71	23.0
Infectious disease	63	20.4
Dermatologist	48	15.5
Neurologist	31	10.0
General internist	33	10.7
Geriatrician	22	7.1
Oncologist	17	5.5
Nurse	24	7.8
level of expertise		
General practitioner	46	14.9
Resident	31	10.0
Specialist	51	16.5
Fellow	15	4.9
Consultant	166	53.7
Experience years		
10 or fewer	158	51.1
11–20	93	30.1
21–30	40	12.9
>30	18	5.8
Practice setting		
Private hospital	58	18.8
Government hospital	177	57.3
Clinic	26	8.4
1ry healthcare	48	15.5
No. of patients		
10 or fewer	25	8.1
11-15	49	15.9
16-20	51	16.5
21-25	70	22.7
>25	114	36.9
Vaccine availability		
No	82	26.5
Yes	227	73.5

HCWs' knowledge regarding RZV

Based on the HCWs' responses to various knowledge questions related to RZV, n = 267 (86.4%) know that the two doses of RZV should be two to six months apart, while n = 205 (66.3%) know that patients should get RZV, even if he/she has already had shingles. However, n = 148 (47.9%) thought that experiencing side effects from the first dose of RZV was a reason not to receive the second dose. Also, n = 146 (47.2%) thought that before administering RZV, the provider must ensure a history of chickenpox or positive VZV serology, and n = 67 (21.7%) do not know that even if a patient experiences side effects from the first dose of RZV, that person should receive the second dose of RZV (Table 2).

**Table 2:** Healthcare workers' knowledge about the zoster vaccine

	Answer	Correct		Incorrect		Don’t know	
The two doses of RZV should be 2-6 months apart.	True	267	86.4%	27	8.7%	15	4.9%
If the recommended interval between RZV doses is exceeded, then the series should be restarted.	False	130	42.1%	135	43.7%	44	14.2%
A patient should get RZV, even if he/she has already had shingles.	True	205	66.3%	56	18.1%	48	15.5%
Before administering RZV the provider must ensure a history of chickenpox or positive VZV serology.	False	120	38.8%	146	47.2%	43	13.9%
If a patient experiences side effects from the first dose of RZV that prevents normal activities, then that person should not receive the second dose of RZV.	False	94	30.4%	148	47.9%	67	21.7%

The relation between HCWs' knowledge and specialty is also addressed. Higher percentages of HCWs from all specialties truly answered the first and third statements, i.e., “the two doses of RZV should be two to six months apart” and “a patient should get RZV, even if he/she has already had shingles,” respectively. Around 50% and more of infectious disease doctors truly answered all the questions, indicating their higher knowledge regarding RZV than other specialties (Table 3).

**Table 3:** Relation between healthcare workers' knowledge and specialty

Specialty	Percentage of correct answers		
	1: The two doses of SHINGRIX should be two to six months apart (correct answer: true).		
Family physician	52	73.2%	
Infectious disease	60	95.2%	
Dermatologist	39	81.3%	
Neurologist	30	96.8%	
General internist	26	78.8%	
Geriatrician	21	95.5%	
Oncologist	17	100.0%	
Nurse	22	91.7%	
	2: If the recommended interval between SHINGRIX doses is exceeded, the series should be restarted (correct answer: false).		
Family physician	21	29.6%	
Infectious disease	39	61.9%	
Dermatologist	11	22.9%	
Neurologist	17	54.8%	
General internist	16	48.5%	
Geriatrician	9	40.9%	
Oncologist	7	41.2%	
Nurse	10	41.7%	
	3: A patient should get SHINGRIX, even if he/she has already had shingles (correct answer: true).		
Family physician	40	56.3%	
Infectious disease	52	82.5%	
Dermatologist	28	58.3%	
Neurologist	24	77.4%	
General internist	21	63.6%	
Geriatrician	15	68.2%	
Oncologist	14	82.4%	
Nurse	11	45.8%	
	4: Before administering SHINGRIX, the provider ensures a history of chickenpox or positive serology (correct answer: false).		
Family physician	27		38.0%
Infectious disease	38		60.3%
Dermatologist	12		25.0%
Neurologist	14		45.2%
General internist	13		39.4%
Geriatrician	4		18.2%
Oncologist	5		29.4%
Nurse	7		29.2%
	5: If a patient experiences side effects from the first dose of SHINGRIX that prevents normal activities, then that person should not receive the second dose of SHINGRIX (correct answer: false).		
Family physician	15		21.1%
Infectious disease	34		54.0%
Dermatologist	14		29.2%
Neurologist	10		32.3%
General internist	5		15.2%
Geriatrician	6		27.3%
Oncologist	3		17.6%
Nurse	7		29.2%

HCWs' attitude regarding RZV

**Figure 1:**
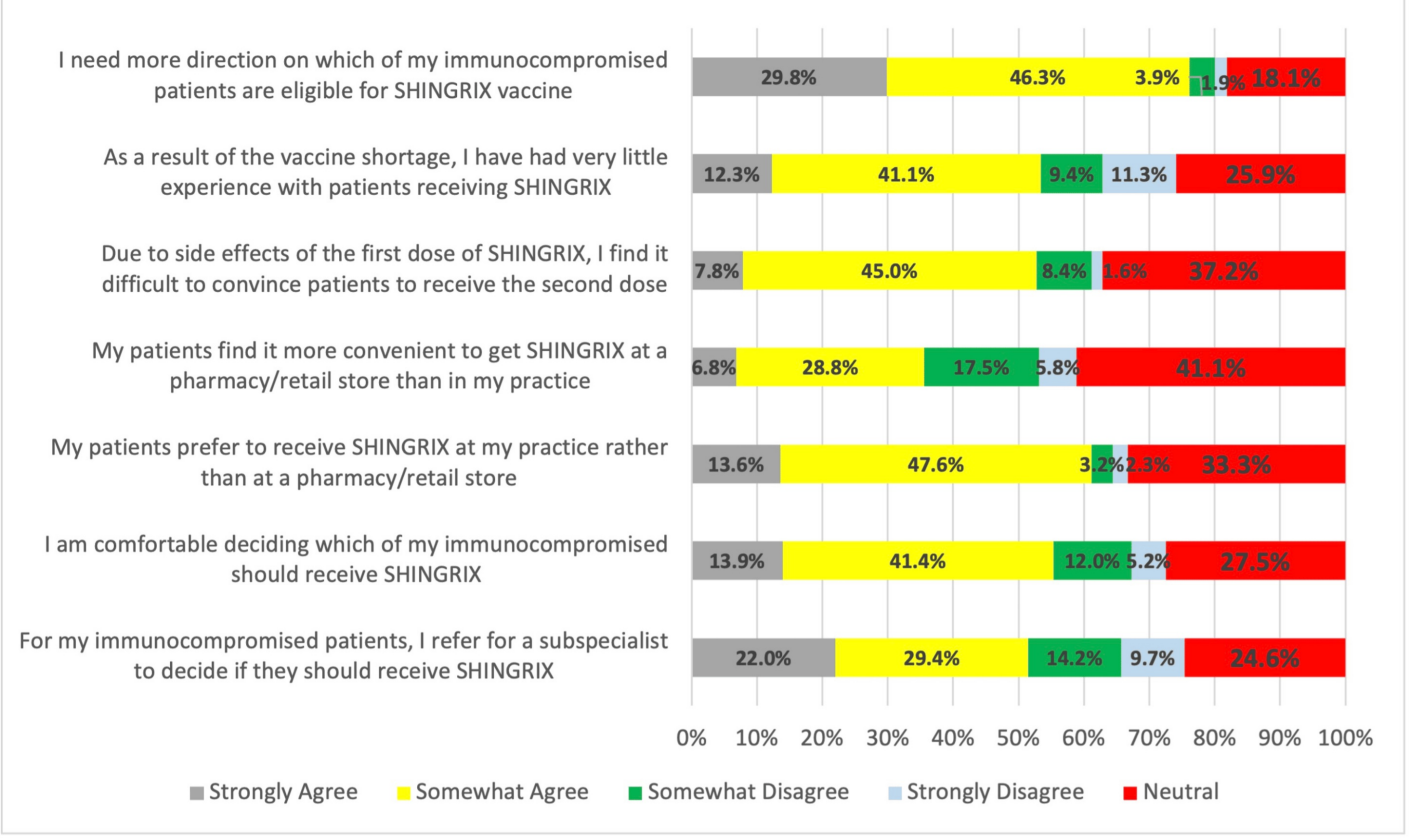
Healthcare workers' attitude toward zoster vaccine

Regarding HCWs' attitudes regarding the zoster vaccine, most of them (n = 235, 76.1%) agreed that they need more direction on which immunocompromised patients are eligible for the RZV vaccine. Some had very little experience with patients receiving RZV due to vaccine shortage (n = 165, 53.4%), and some also referred to a subspecialist to decide if they should receive RZV (n = 159, 51.4%). Moreover, n = 189 (61.2%) agreed that their patients prefer to receive RZV at the practice rather than a pharmacy/retail store (Figure 1).

HCWs' practices regarding RZV

**Figure 2:**
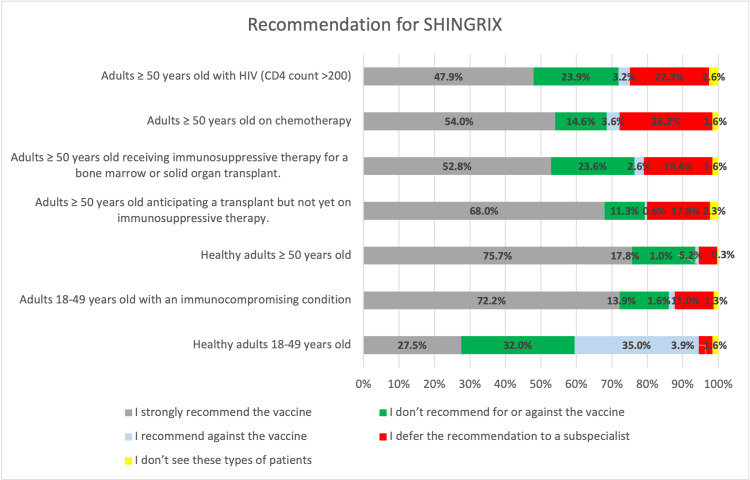
Healthcare workers' practices regarding the zoster vaccine

HCWs' recommendations for RZV in various patient groups are presented. Most of the respondents follow ACIP recommendations for the vaccine. Among the respondents, n = 234 (75.7%) recommend the vaccine for healthy adults ≥50 years old, n = 223 (72.2%) recommend the vaccine for adults 18-49 years old with an immunocompromised condition, n = 210 (68%) recommend the vaccine for adults ≥50 years old anticipating a transplant but not yet on immunosuppressive therapy, n = 167 (54.0%) recommend the vaccine for adults ≥50 years old on chemotherapy, and n = 163 (52.8%) recommend the vaccine for adults ≥50 years old receiving immunosuppressive therapy for a bone marrow or solid organ transplant. However, n = 85 (27.5%) recommended the vaccine for healthy adults 18-49 years old (Figure 2).

The HCWs used different methods to bring patients back for their second dose. In particular, n = 142 (46%) rely on the patient to remember to come back to receive the second dose, n = 141 (45.6%) refer patients to the vaccine manufacturer for their reminder/recall system, n = 118 (38.2%) conduct a reminder/recall for patients to come back for the second dose, and n = 74 (23.9%) give patients an appointment to return and receive the second dose (Table 4). 

**Table 4:** Methods used to bring patients back for their second dose of SHINGRIX

	Yes		No	
We give patients an appointment to return and receive the second dose	74	23.9%	235	76.1%
We rely on the patient to remember to come back to receive the second dose	142	46.0%	167	54.0%
We conduct reminder/recall (mail, e-mail, phone or text, etc.) for patients to come back and receive the second dose	118	38.2%	191	61.8%
We refer them to the vaccine manufacturer for their reminder/recall system	141	45.6%	168	54.4%

HCWs' experience with patients declining RZV

**Figure 3:**
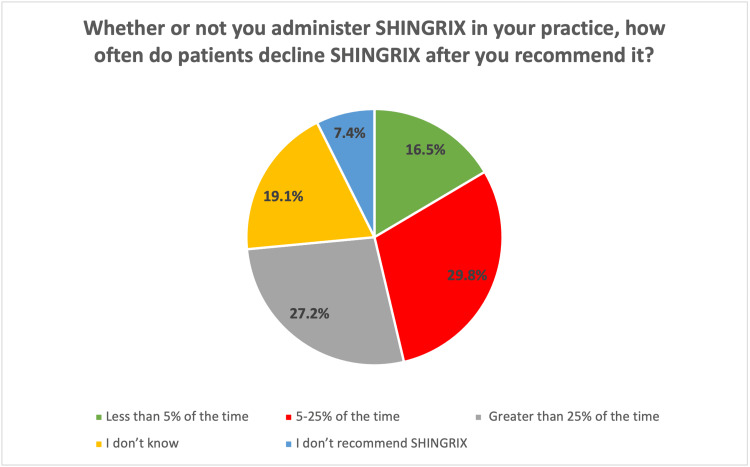
Percentage of patients declining zoster vaccine following recommendation

Following the recommendation of zoster vaccine, n = 84 (27.2%) of HCWs reported that patients declined VZV >25% of the time, n = 92 (29.8%) reported that patients declined 5-25%, and n = 51 (16.5%) reported that patients declined 5% of the time after it is recommended (Figure 3).

**Figure 4:**
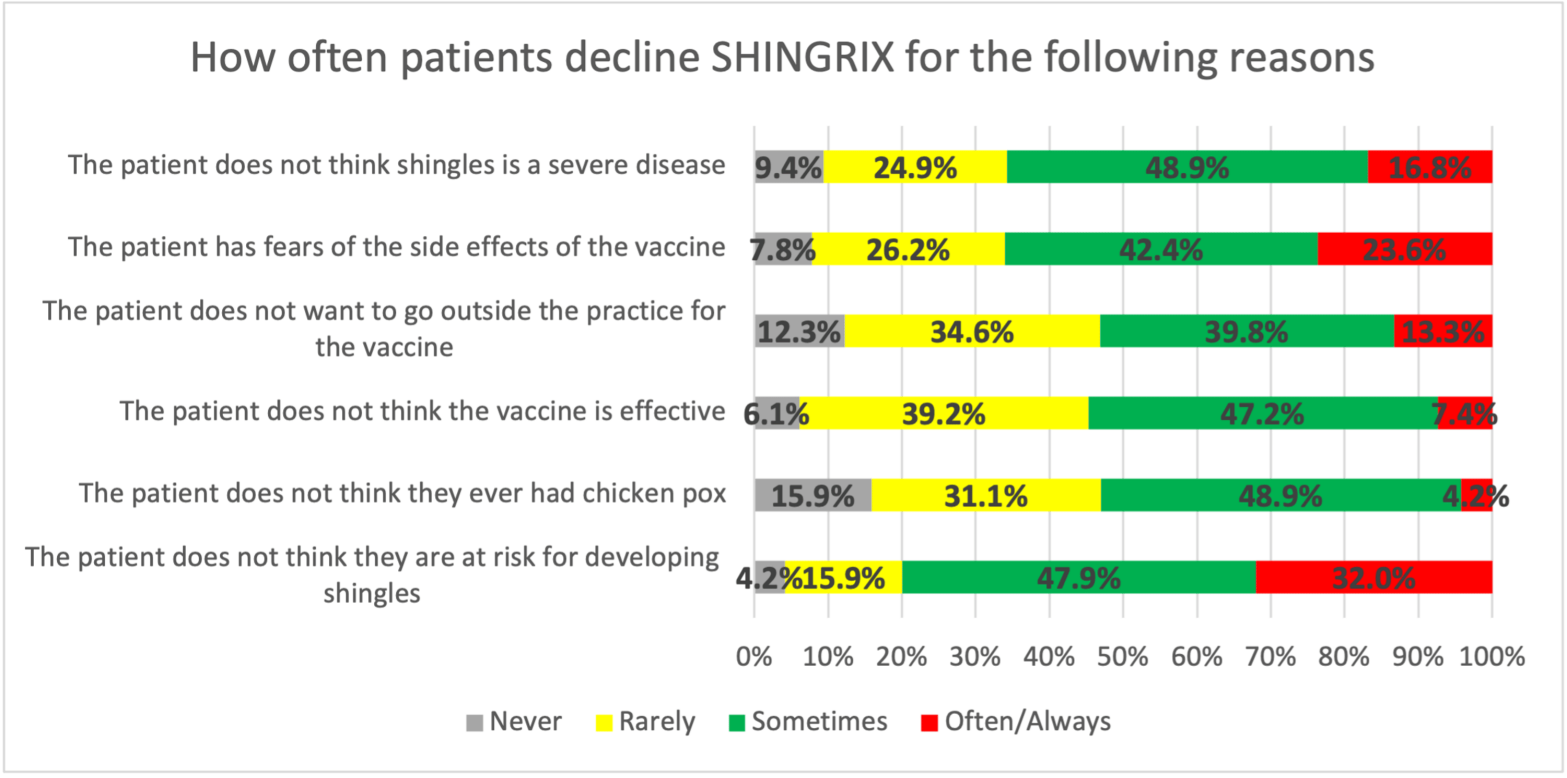
Healthcare workers' experience with patients declining zoster vaccine

Physicians reported that patients most frequently decline RZV as they do not think they are at risk for developing shingles (n = 99, 32% "often/always"; n = 148, 47.9% "sometimes"), fears of side effects (n = 73, 23.6% "often/always”; n = 131, 42.4% "sometimes”), and patients don’t think shingles is a severe disease (n = 52, 16.8% “often/always”, n = 151, 48.9% “sometimes”) (Figure 4).

HCWs reported barriers to recommending RZV

The most commonly reported barriers to recommending RZV included patients having a severe reaction to the first dose (n = 51, 16.5% "major”; n = 82, 26.5% “moderate” barrier), the limited supply of RZV (n = 31, 10% “major"; n = 123, 39.8% “moderate” barrier), RZV has not been studied enough in immunosuppressed patients (n = 21, 6.8% “major”; n = 79, 25.6% “moderate” barrier), and more pressing medical issues taking precedence (n = 12, 3.9% “major”; n = 94, 30.4% “moderate” barrier) (Table 5).

**Table 5:** Barriers toward zoster vaccine

	Not a barrier		Minor barrier		Moderate barrier		Major barrier	
Limited supply of RZV	60	19.4%	95	30.7%	123	39.8%	31	10.0%
More pressing medical issues take priority, I often forget to discuss RZV with my patients	48	15.5%	155	50.2%	94	30.4%	12	3.9%
My concerns about safety of RZV	104	33.7%	117	37.9%	79	25.6%	9	2.9%
My concerns about side effects of RZV	83	26.9%	145	46.9%	63	20.4%	18	5.8%
The fact that two doses of RZV are required	103	33.3%	107	34.6%	87	28.2%	12	3.9%
RZV has not been studied enough in immunosuppressed patients	109	35.3%	100	32.4%	79	25.6%	21	6.8%
My patients having a severe reaction to the first dose of RZV	85	27.5%	91	29.4%	82	26.5%	51	16.5%

HCWs' strategies to increase vaccine uptake

**Figure 5:**
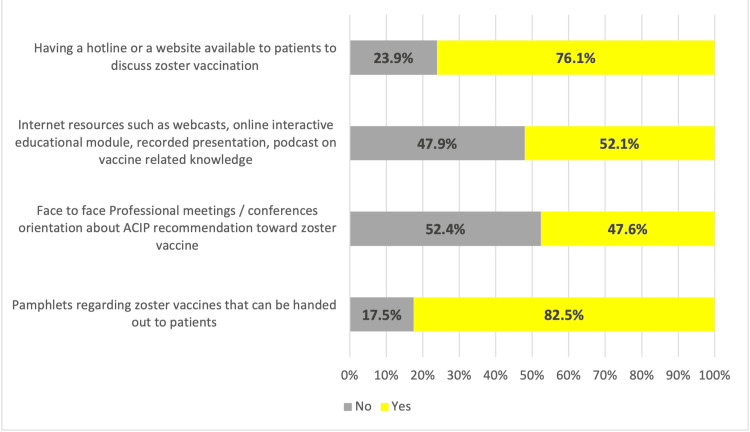
Strategies to increase uptake of zoster vaccine

A total of 255 (82.5%) HCWs chose the pamphlets regarding zoster vaccines that can be handed out to patients; n = 235 (67.1%) recommend having a hotline or a website available to patients to discuss zoster vaccination; n = 161 (52.1%) recommend Internet resources such as webcasts, online interactive educational modules, recorded presentations, and podcasts on vaccines; and n = 147 (47.6%) chose face-to-face professional meetings and conferences orientation about ACIP recommendation toward zoster vaccine (Figure 5).

## Discussion

To our knowledge, this represents the first assessment of the physicians’ perspective of RZV in Saudi Arabia following ACIP recommendations for licensure in 2021. This questionnaire study was administered to 309 HCWs who care for people with a higher risk of HZ infection and complications to recognize HCWs' KAP on HZ vaccination.

The survey findings reveal a mixed level of knowledge and varied practices regarding HZ vaccination among HCWs. The HCWs exhibited a relatively high level of knowledge about key aspects of the RZV, although some gaps remain. A significant 86.4% of HCWs correctly identified that the two doses of RZV should be spaced two to six months apart, and 66.3% knew that individuals who have had shingles should still receive the vaccine. These findings are consistent with recommendations from ACIP, which state that the RZV vaccine schedule involves two doses given two to six months apart and that individuals who have previously had shingles should still be vaccinated to reduce the risk of recurrence. However, the study also highlights gaps in HCWs’ understanding. For instance, 47.9% of the respondents incorrectly believed that side effects from the first dose were a valid reason not to receive the second dose. This knowledge gap is concerning because it could result in patients failing to complete the two-dose regimen, which is essential for optimal protection against shingles. Leigh et al. found that vaccine hesitancy due to concerns about side effects is a common barrier to vaccine uptake, particularly when healthcare providers themselves are unsure about the safety or necessity of completing a full vaccination course [29]. This is also consistent with findings from the study done by Yawn et al., which noted reluctance among pulmonologists to continue vaccination when patients report side effects [27]. Another notable gap is the belief held by 47.2% of HCWs that a history of chickenpox or positive varicella-zoster virus (VZV) serology is required before administering RZV. This misconception could lead to unnecessary delays in vaccination or missed opportunities for protection. According to Lin KY et al., it is not mandatory to confirm prior infection with chickenpox or perform VZV serology before administering RZV, as the vaccine is recommended for all individuals aged 50 years and older, regardless of history of shingles or chickenpox [30]. This indicates a need for better education about vaccine indications and contraindications. Singer D et al. reported also a knowledge gap in staff regarding the zoster vaccine where only 43% of specialists correctly answered all vaccine-related questions [31]. On the contrary, physicians were generally knowledgeable about RZV in a study done by Hurley et al. [15].

The difference in knowledge between specialties is also an interesting finding. The study reports that infectious disease specialists exhibited higher knowledge about RZV than other HCWs. This aligns with the findings of Wie SH et al., who noted that specialists in infectious diseases or immunology were more likely to be well-versed in the latest vaccine guidelines and to encourage vaccination in their patient populations [32]. The primary care providers or general practitioners reported to have less frequent exposure to vaccine-specific guidelines, and additional training and resources should be provided to close these knowledge gaps [33].

HCWs’ attitude toward RZV, as reported in this study, revealed a mixed response. The majority (76.1%) agreed that they need more guidance on which immunocompromised patients are eligible for RZV, suggesting a need for clearer guidelines or additional training on vaccinating high-risk populations. Peterson et al. also found that HCWs often feel uncertain about vaccinating immunocompromised patients, particularly in light of concerns about vaccine safety and efficacy in these groups [34]. In Hurley et al., a similar finding emerged, with a majority of family physicians indicating a need for guidance on patient eligibility for RZV [15]. As immunocompromised patients are at higher risk of shingles, targeted education is crucial to ensure that HCWs feel confident in recommending and administering the vaccine to these individuals.

The finding that 53.4% of HCWs had limited experience with administering RZV due to vaccine shortage in the private sector is consistent with the ongoing challenges faced by the healthcare system in maintaining an adequate supply of vaccines in all healthcare sectors. Wiot et al. also noted that vaccine shortage can create frustration among healthcare providers, potentially leading to delays in vaccination and reduced uptake [35]. This issue underscores the importance of improving vaccine distribution systems to ensure timely access to vaccines, particularly for high-risk populations who are more susceptible to complications from shingles in all healthcare facilities.

Moreover, the fact that 51.4% of HCWs referred patients to a subspecialist for vaccination decisions also points to a reluctance to make independent decisions about RZV in some healthcare settings. This trend is supported by research from Lin et al., which found that many healthcare providers defer vaccination decisions to specialists due to perceived uncertainty or a lack of familiarity with complex vaccination guidelines [36]. This suggests a need for increased confidence-building among HCWs, particularly in general practice settings, to empower them to make vaccination recommendations on their own.

In addition, 61.2% of HCWs agreed that patients prefer to receive RZV in their primary care practice rather than at a pharmacy or retail store. This finding reflects patients’ desire for convenience and trusted healthcare provider involvement in vaccine administration, as supported by Ahmed et al.'s study [37]. Offering vaccines within the primary care setting ensures that patients receive trusted medical advice and are more likely to complete the vaccine series, which can improve overall vaccination rates.

The study reflects that most healthcare providers follow the ACIP recommendations for RZV across patient demographics. For instance, high percentages of healthcare providers recommend RZV for healthy adults over 50 (75.7%) and adults aged 18-49 with immunocompromising conditions (72.2%). This adherence is consistent with findings in the article of Hurley et al., which shows that a significant portion of primary care physicians already recommends RZV to immunocompromised individuals, particularly those ≥50 with conditions like HIV or on immune-modulating treatments [15]. According to a study done by Dooling KL et al., HCWs' recommendation practices often reflect guidelines provided by health authorities like the CDC and ACIP, which in turn influences the willingness of patients to accept the vaccine [38]. While adherence is strong, some differences arise in the recommendations for specific groups. For example, 27.5% of HCWs in this study recommended the vaccine for healthy adults 18-49 years old. Similarly, Singer et al. noted that while a majority recognize herpes zoster as a risk and value RZV, specialists’ knowledge about all ACIP indications is low [31].

Regarding the strategies for ensuring patients receive their second dose of RZV, the current study reports that many healthcare systems rely on patient self-reporting with minimal follow-up measures, which can be ineffective in ensuring adherence. George et al. highlighted that using a more structured follow-up system (such as setting an appointment for the second dose or providing direct reminders) improves completion rates for vaccines like RZV [39]. The relatively low use of appointment setting (23.9%) in the study is concerning and reflects broader trends in healthcare practice, where time and resource limitations can reduce the effectiveness of follow-up strategies.

The study shows that a substantial proportion of patients decline the RZV, due to perceived low risk of shingles, fears of side effects, or doubts about the severity of the disease, which is consistent with findings in other studies. For example, Wang et al. found that many patients overestimate their personal immunity or underestimate the risks of shingles, which leads to vaccine hesitancy [40]. They also highlighted that education about the potential complications of shingles, such as post-herpetic neuralgia, is often insufficient. This supports the finding of our study that many patients do not see shingles as a severe disease (16.8% “often/always,” 48.9% “sometimes”) and therefore do not prioritize vaccination. A similar opinion was noted by AlMuammar et al. where the top reasons for vaccine refusal were related to misconceptions about the risk of shingles, concerns over side effects, and a belief that the vaccine wasn’t necessary for healthy individuals [41]. Alamri et al. reported that perceived side effects, particularly pain or swelling at the injection site, were common deterrents among vaccine recipients [42]. The study by López-Fauqued M et al. further supports this, noting that while the safety profile of RZV is generally positive, concerns about side effects often fear the patients, leading to significant declines in vaccination offers [43].

The barriers that HCWs face in recommending RZV, particularly the concerns about patient reactions to the first dose, the limited vaccine availability, and the perception that RZV has not been adequately studied in immunocompromised patients, are well-documented in the literature. Peterson et al. found that adverse reactions following the first dose of RZV were a significant concern among healthcare providers, which may explain the hesitancy to recommend the vaccine to certain groups [34]. This aligns with the findings in the present study, where 16.5% of HCWs described adverse reactions to the first dose as a major barrier. However, it is important to note that while side effects are a legitimate concern, studies such as Parikh et al., have shown that the risk of severe reactions is relatively low, and most adverse events are mild and transient [44]. Limited vaccine availability is another barrier, with 10% of HCWs citing this as a “major” obstacle. This issue was also identified by Iwu-Jaja et al. who reported that vaccine shortages, particularly during peak vaccination seasons, can lead to delays in patient vaccinations and reduced overall uptake [45]. The authors suggested that improving the supply chain for vaccines, increasing inventory management, and ensuring timely distribution could help alleviate this issue. Regarding immunocompromised patients, the concerns expressed by HCWs (6.8% major, 25.6% moderate) about the adequacy of RZV studies in these populations are well-founded, as there has historically been limited data on the safety and efficacy of vaccines in immunocompromised individuals. Stefanizzi et al. noted that while RZV is recommended for immunocompromised patients, some HCWs remain hesitant due to a lack of long-term data on how the vaccine performs in these vulnerable groups [46]. However, more recent studies, including the one by Omari et al., have shown that RZV is safe and effective in immunocompromised patients, suggesting that this concern may gradually decrease with greater dissemination of updated evidence [47].

The strategies identified for increasing RZV uptake, such as providing educational pamphlets (82.5%), using hotlines or websites (67.1%), and offering online resources (52.1%), reflect a growing recognition of the importance of patient education and accessible information. A study by Almakhdob et al. found that educational outreach is one of the most effective methods for improving vaccine uptake, especially when combined with patient-centered communication strategies [48]. These findings align with the data provided in the study, where pamphlets and online resources were chosen as primary methods for promoting RZV.

The role of professional meetings and conferences in educating HCWs (47.6%) is also significant, as continuous professional development has been shown to improve vaccination recommendation rates. George et al. highlighted that face-to-face meetings and training sessions are particularly effective in ensuring that healthcare providers remain updated on the latest vaccine guidelines and best practices for patient communication [39].

Finally, AlMuammar et al. found that reminder and recall systems, including phone calls, text messages, and automated systems, significantly improved vaccination completion rates [41]. This supports the finding in our study that relying on patients to remember to return for their second dose was less effective compared to structured reminder systems. Integrating patient appointment scheduling with reminder systems may further enhance adherence rates.

While this survey study includes samples of HCWs in Saudi Arabia, certain limitations must be acknowledged. The findings are based on self-reported data rather than direct observation of actual practice or qualitative responses. In addition, physicians from smaller practices are slightly underrepresented. Moreover, our results do not clarify whether some physicians genuinely oppose vaccine use in immunocompromised patients or if they need more direction or prefer to defer the decision to a subspecialist.

## Conclusions

Overall, HCWs demonstrated an understanding of the key recommendations for RZV, but still, there are areas, especially in vaccine indications and contraindications, where knowledge gaps exist. By addressing these gaps through targeted education and professional development, healthcare systems can increase confidence in recommending RZV. Moreover, fostering a positive attitude toward vaccination, particularly regarding immunocompromised patients and overcoming vaccine shortages, will be essential in achieving higher vaccination rates and reducing the incidence of shingles and its complications.

The main challenges toward vaccine uptake include patient misconceptions about the risk of shingles, concerns about side effects, and logistical barriers such as vaccine availability and inadequate follow-up systems. The strategies recommended by HCWs, including educational initiatives and digital resources, align with evidence-based practices that have been shown to improve vaccine uptake. By addressing these barriers and implementing comprehensive, multi-channel strategies for increasing HCWs' awareness, patient education, and follow-up, healthcare systems can increase RZV vaccination rates and reduce the burden of shingles, particularly in high-risk populations.

## Appendices

Study questionnaire

Dear participants,

Thank you for taking the time to complete this questionnaire. We are fourth-year medical students at Dar Al Uloom University College of Medicine conducting a cross-sectional questionnaire-based study to assess healthcare workers' knowledge, attitude, and practice toward the shingles vaccine.

Please read the following before answering the questionnaire:

For the prevention of shingles, the Centers for Disease Control and Prevention recommended SHINGRIX (recombinant zoster vaccine) in 2018 for adults ³ 50 years. In October 2021, the Advisory Committee on Immunization Practice (ACIP) recommended SHINGRIX use in immunocompromised adults aged ≥ 18 years.

A. Demographic data and practice characteristics

1. Gender

Male

Female

2. Age in years

25-34

35-44

45-54

55-65

3. Specialty

Family physician

Infectious disease

Dermatologist

Neurologist

General internist

Geriatrician

Oncologist

Nurse

Other (please specify)

4. Level of expertise

General practitioner

Resident

Specialist

Fellow

Consultant

5. Experience years

10 or fewer

11-20

21-30

>30

6. Practice setting

Private hospital

Government hospital

Clinic

1ry healthcare

7. No of patients seen/day

10 or fewer

11-15

16-20

21-25

>25

8. Zoster vaccine availability in your healthcare

Yes

No

B. Healthcare workers' knowledge about zoster vaccine

1. Based on your current knowledge and without consulting other sources for information, please answer whether each of the following statements is true, false, or you don’t know.

The two doses of SHINGRIX should be two to six months apart.

If the recommended interval between SHINGRIX doses is exceeded, then the series should be restarted.

A patient should get SHINGRIX, even if he/she has already had shingles.

Before administering SHINGRIX the provider must ensure a history of chicken pox or positive VZV serology.

If a patient experiences side effects from the first dose of SHINGRIX that prevents normal activities, then that person should not receive the second dose of SHINGRIX.

C. Healthcare workers' attitude toward zoster vaccine

1. Please tell us how strongly you agree or disagree with each of the following statements about the delivery of SHINGRIXÒ to your adult patients. (Please check the ONE best response for each row)

a. For my immunocompromised patients, I refer a subspecialist to decide if they should receive SHINGRIX.

b. I am comfortable deciding which of my immunocompromised should receive SHINGRIX.

c. My patients prefer to receive SHINGRIX at my practice rather than at a pharmacy/retail store.

d. My patients find it more convenient to get SHINGRIX at a pharmacy/retail store than in my practice.

e. Due to the side effects of the first dose of SHINGRIX, I find it difficult to convince patients to receive the second dose.

f. As a result of the vaccine shortage, I have had very little experience with patients receiving SHINGRIX.

g. I need more direction on which of my immunocompromised patients are eligible for the SHINGRIX vaccine.

D. Healthcare workers' practices regarding zoster vaccine

1. For each type of patient below, which answer best describes your recommendation for SHINGRIX? (Please check the ONE best response for each type of patient).

a. Healthy adults 18-49 years old

b. Adults 18-49 years old with an immunocompromising condition

c. Healthy adults ≥ 50 years old

d. Adults ≥ 50 years old anticipating a transplant but not yet on immunosuppressive therapy.

e. Adults ≥ 50 years old receiving immunosuppressive therapy for a bone marrow or solid organ transplant.

f. Adults ≥ 50 years old on chemotherapy

g. Adults ≥ 50 years old with HIV (CD4 count >200)

2. Which of the following methods do you use to bring patients back for their second dose of SHINGRIXÒ? (Please answer “Yes” or “No” for each statement)

a. We give patients an appointment to return and receive the second dose.

b. We rely on the patient to remember to come back to receive the second dose.

c. We conduct reminder/recall (mail, e-mail, phone or text, etc.) for patients to come back and receive the second dose.

d. We refer them to the vaccine manufacturer for their reminder/recall system.

E. Healthcare workers' experience with patients declining zoster vaccine.

1. Whether or not you administer SHINGRIX in your practice, how often do patients decline SHINGRIX after you recommend it?

Less than 5% of the time

5-25% of the time

Greater than 25% of the time

I don’t know

I don’t recommend SHINGRIX

2. Please tell us how often patients decline SHINGRIX for the following reasons.

(Please check the ONE best response for each row)

a. The patient does not think they are at risk for developing shingles.

b. The patient does not think they ever had chicken pox.

c. The patient does not think the vaccine is effective.

d. The patient does not want to go outside the practice for the vaccine.

e. The patient has fears of the side effects of the vaccine.

f. The patient does not think shingles is a severe disease.

F. Barriers toward vaccine and strategies to increase uptake

1. Whether or not you currently administer SHINGRIX in your practice, please tell us how much each of the following is a barrier for you to recommend SHINGRIX to your eligible patients.

(Please check the ONE best response for each barrier)

a. Limited supply of SHINGRIX

b. More pressing medical issues take priority, I often forget to discuss SHINGRIX with my patients.

c. My concerns about the safety of SHINGRIX

d. My concerns about the side effects of SHINGRIX

e. The fact that two doses of SHINGRIX are required.

f. SHINGRIX has not been studied enough in immunosuppressed patients.

g. My patient had a severe reaction to the first dose of SHINGRIX.

2. Which of the following would help to increase zoster vaccine uptake? (choose all apply)

a. Face to face Professional meetings/conferences orientation about ACIP recommendation toward zoster vaccine

b. Internet resources such as webcasts, online interactive educational modules, recorded presentations, and podcasts on vaccine-related knowledge

c. Having a hotline or a website available to patients to discuss zoster vaccination

d. Pamphlets regarding zoster vaccines that can be handed out to patients
